# Compatibility optimization of the traditional Chinese medicines ‘Eczema mixture’ based on back-propagation artificial neural network and non-dominated sorting genetic algorithm

**DOI:** 10.3389/fphar.2025.1593783

**Published:** 2025-05-02

**Authors:** Xin He, Zhijie Song, Yanqun Yang, Siqi Wu, Shuo Meng, Huanyu E, Hongfei Li, Guoyu Ding

**Affiliations:** ^1^ Department of Medical Oncology, The First Hospital of China Medical University, Shenyang, China; ^2^ School of Pharmacy, Shenyang Medical College, Shenyang, China; ^3^ Shenyang 15th Retired Cadres’ Center, Liaoning Province Military Command, Shenyang, China

**Keywords:** Chinese medicine formula, TCM compatibility, multi-objective optimization, artificial neural network, non-dominated sorting genetic algorithm

## Abstract

**Introduction:**

Chinese medicine formulas (CMF) are an important aspect of traditional Chinese medicine (TCM) and are formulated based on strict compatibility proportions guided by TCM theory. Due to the complex chemical constituents of TCM and the diversity of evaluation indicators for a certain disease, the research strategy on how to obtain the optimal combination of these crude extracts, homologous compounds or even the specific compounds mixture becomes the key step in the study of compatibility proportion research. Therefore, in this research, the “Eczema mixture” (EM) which includes six kinds of Chinese medicinal materials for the treatment of atopic dermatitis was cited as an example to illustrate the proposed compatibility optimization strategy.

**Methods:**

Ultra-performance liquid chromatography-quadrupole/time-of-flight (UPLC-Q/TOF) technology was used to analyze the chemical components in the EM formula, and a total of 136 chemical compounds were identified. 76 formulas with different compatibility ratios were generated with the simplex centroid mixture design (SCMD). Two defined objective functions, the maximum of the anti-inflammatory and anti-allergic activity were used to evaluate the bioactivities of all the formulas. The 6-n-2 three-layers of back-propagation artificial neural network (BP-ANN) was employed to model the two defined objective functions. With the predictive models, the Pareto front was determined by a variant of non-dominated sorting genetic algorithm II(VNSGAII) to provide the optimal prescription set.

**Results:**

The 6-n-2 three-layers of artificial neural networks demonstrated a satisfactory fitting effect for the nonlinear activity relationship. In the EM formula, Huangbai and Kushen were identified as the main botanical drugs with anti-inflammatory and anti-allergic roles. The results were consistent with the clinical application of the 113 prescriptions involving 230 botanical drugs for the treatment of AD from the ‘Dictionary of Traditional Chinese Medicine Prescription’.

**Conclusion:**

The proposed SCMD-ANN-VNSGAII is a powerful approach that may facilitate future compatibility optimization of homologous compounds or specific component mixtures.

## 1 Introduction

For a long history, Chinese medicine formula (CMF) has been used to treat various diseases under the guidance of traditional Chinese medicine (TCM) theory. Recent advancements in basic research have made significant progress in identifying active chemical components of TCM used in clinical practice (Mukhopadhyay et al., 2023; Krishna et al., 2024). However, the identification and testing of those active ingredients are just the beginning, and there is increasing focus on the synergistic relationship between herbal medicines for disease treatment (Xu et al., 2021; Hou et al., 2024; Lin et al., 2024).

In this research, the ‘Eczema mixture’, a formula consisting of six botanical drugs (*Phellodendri Chinensis* Cortex (Huangbai), *Sophorae Flavescentis* Radix (Kushen), *Honeysuckle* Buds (Jinyinhua), *Sanguisorbae* Radix (Diyu), *Glycyrrhizae Radix Et Rhizoma* (Gancao) and *Schizonepetae* Herba (Jingjie)) from the book ‘Trauma Science of Traditional Chinese Medicine’, was used to optimize and identify the best compatible formula for treating atopic dermatitis (AD) (Group, 1980). AD is a common recurrent allergic and inflammatory skin disease characterized by severe itching and erythema, significantly impacting the quality of life for 30% of children and 10% of adults. As usual, the anti-inflammatory and anti-allergic activities of herbal extracts were used together to evaluate the effects of AD treatment (Park et al., 2020; Min et al., 2021).

For prescription optimization, the choice of optimization methods is crucial. Previous reports have compared several commonly used optimization methods, including the ‘Feedback System Control’, ‘Orthogonal Design’, ‘Uniform Design’, ‘Baseline Isometric Regulation Design’, and ‘Experimental Design (ED) - Nonlinear Modeling (NM) - Multi-objective Optimization (MO) trigeminy method' (Luan et al., 2020). Among these methods, the ‘ED-NM-MO trigeminy method’ has shown no obvious shortcomings except for its complicated calculation process. This method has been previously used for the optimization of *Ginseng Radix et Rhizoma* - *Aconiti Lateralis Radix Praeparata* herbal pair against heart failure (Li et al., 2023) and *Astragali Radix* - *Angelicae Sinensis* Radix herbal pair on proliferation of vascular smooth muscle cells (Chen et al., 2023). The ED-NM-MO method is very suitable for the investigation of medicinal properties and can be used to optimize the dosage and ratio of CMF. Meanwhile, it is also a flexible integrated strategy that involves three steps, and there are many different ways to approach each of these steps. However, the NM stage of the ED-NM-MO trigeminy method generally used the partial least squares (PLSR) algorithm which was not ideal in fitting some complex nonlinear relationships. And the MO stage generally used the simple addition of multiple single target values, leading to a diminished objectivity in multi-objective optimization scenarios. Consequently, it is urgent to update this strategy by incorporating recently introduced innovative fitting and optimization techniques to enhance its capabilities.

In this research, the ED stage of the aforementioned trigeminy method employed SCMD which belongs to the category of mixture design. A key feature of SCMD is its ability to ensure that each formulated mixture maintains a constant total amount. After the ED process, a total of 76 prescriptions with different ratios of botanical drugs were obtained. The anti-inflammatory and anti-allergic activities of these 76 prescriptions were tested. In the NM stage of the ED-NM-MO trigeminy method, a three-layer BP-ANN model was applied to build the non-linear fitting model of different herb combinations and two bioactivities. BP-ANN is a common mathematical tool used for association analysis of TCM formulas. It is suitable for solving nonlinear and nonparametric problems and can approximate any continuous function with desired accuracy (Tang et al., 2022). To build an ideal model, the number of nodes in the hidden layer, the methods of weights and bias values updating, and the learning rate were screened using the TensorFlow platform. Furthermore, another two machine learning algorithms, the random forest regression (RF) and support vector regression (SVR) were compared with BP-ANN in the NM stage of the ED-NM-MO trigeminy method.

Since two bioactivities were used to evaluate the EM formulas, a suitable multi-objective optimization was needed. Here, VNSGAII, as one of the most effective multi-objective genetic algorithms was introduced, which can supply many Pareto optimal solutions for researchers to help them ‘have their cake and eat it’. In the Pareto front solution plane, each point represents a formula with a specific composition. Researchers can select the appropriate point (including the extreme points or the knee point) from them according to clinical requirements.

In this research, the ED-NM-MO trigeminy method was followed, incorporating several updates to different stages of the methodology. The methodological innovation lies in our pioneering implementation of the TensorFlow platform to optimize an artificial neural network (ANN) model for modeling the complex relationship between traditional Chinese medicine (TCM) compound ratios and bioactivity. By leveraging the platform’s hyperparameter search performance through an exhaustive search across all possible parameter combinations, we identified optimal values for critical model parameters including: the number of hidden layer nodes, learning rate, dropout rate, and parameter update strategies, to achieve model optimization. Indeed, based on our research,the integration of artificial neural networks (ANN) with hyperparameter search demonstrated superior performance compared to conventional machine learning algorithms such as Random Forest and Support Vector Regression (SVR), particularly in handling complex nonlinear relationships inherent in TCM formulation-activity modeling. Meanwhile, the proposed SCMD-ANN-VNSGAII methodology not only updated the trigeminy strategy but also provided credible results for the compatibility optimization of the EM formula. This methodology has the potential to pave the way for future compatibility optimization of homologous compounds or specific component mixtures.

## 2 Materials and methods

### 2.1 Chemicals and reagents

Dulbecco’s modified Eagle’s medium (DMEM) was purchased from Gibco BRL (Grand Island, NY, United States). Fetal bovine serum (FBS) was obtained from Biowest (Kansas City, MO, United States). 3-(4,5-Dimethylthiazol-2-yl)-2,5-diphenyltetrazolium bromide (MTT), LPS, p-nitropheny-N-acetyl-β-D-glucosaminide, PMA and A23187 were supplied by Sigma-Aldrich (St. Louis, MO, United States). Rat basophilic leukemia (RBL-2H3, ATCC^®^ CRL-2256) and murine macrophage (RAW264.7, ATCC^®^ TIB-71) cells were procured from the American Type Culture Collection (ATCC).

Six kinds of Chinese medicinal materials (Huangbai, Diyu, Kushen, Gancao, Jinyinhua and Jingjie) were purchased from AnGuo herb markets in China. All of these samples were identified by Professor Junping Wang from Shenyang Medical College. And voucher specimens of the above six botanical drugs were deposited in the Specimen Museum of the School of Traditional Chinese Medicine, Shenyang Medical University.

### 2.2 Simplex centroid mixture design

The SCMD was applied to define the optimum mixture proportion of these six selected Chinese medicinal components. Supplementary Table S1 presents a matrix design consisting of 76 experimental points. These six independent variables in the mixture design, which were Huangbai, Diyu, Kushen, Gancao, Jinyinhua and Jingjie, were studied at 9 levels: 0, 1/12 × 50, 1/6 × 50, 1/5 × 50, 1/4 × 50, 1/3 × 50, 1/2 × 50, 7/12 × 50 and 1 × 50. These two dependent variables were anti-inflammatory and anti-allergic effects. All the mixtures in the matrix must have the same final weight of 50 mg. The differences between prescriptions were in the proportion of medicinal materials. Due to the large number of experiments, the blocks were set as 3 and 6 augment designs were introduced. The experiment design was performed using Design Expert 11 software (Version 11.0.4.0, Stat-Ease Inc., 2021 East Hennepin Ave., Suite 480 Minneapolis, MN 55413).

### 2.3 Sample preparation

In this study, six kinds of Chinese medicinal materials were finely pulverized and filtered through a 100-mesh sieve to obtain a uniform particle size. The mixtures in the prescriptions, each weighing 50 mg, were extracted with 5 mL of a methanol-water (5:5, v/v) solution. The extraction process was carried out using an ultrasonic extraction apparatus operating at a frequency of 40 kHz and a power of 500 W. The extraction was performed for 30 min at room temperature. After the extraction, the samples were centrifuged at 12,000 rpm for 10 min. The supernatant was volatilized and freeze-dried to obtain the dry powder.

### 2.4 UPLC/Q-TOF-MS analysis

For the sample analysis, a Waters Acquity UPLC system (Waters Co., Milford, MA, United States) was used. The ionized samples were prepared using a Waters Q-TOF Premier Mass Spectrometer with an electrospray ionization system (Water MS Technologies, Manchester, UK). Data acquisition was performed using MassLynx V4.1 software (Waters Co., United States). A Waters ACQUITY UPLC BEH C_18_ column (100 mm × 2.1 mm, 1.7 μm) was used for the separation of compounds at 25 °C. The mobile phase consisted of acetonitrile (A) and water with 1% formic acid (B) at a flow rate of 0.4 mL min^-1^. Gradient elution was completed as follows: 0–5 min, 98%–98% (v/v) A; 5–9 min, 98%–92% (v/v) A; 9–12 min, 92%–92% (v/v) A; 12–15 min, 92%–90% (v/v) A; 15–40 min, 90%–40% (v/v) A; 40–42 min, 40%–20% (v/v) A; 42–45 min, 20%–98% (v/v) A; 45–48 min, 98%–98% (v/v) A.

### 2.5 Cell viability assay

Cell viability was assessed using the MTT assay, as described in reference (Boonyong et al., 2023). RAW264.7 and RBL-2H3 cells were seeded into 96-well plates at a density of 10^4^ cells per well for 12 h before treatment. The cells were then incubated with various concentrations of single medicinal materials from EM (0.2–2 mg/mL for RBL-2H3; 0.1–0.7 mg/mL for RAW264.7) for 24 h. After the incubation period, the cells were incubated with 5 mg/mL MTT for an additional 4 h at 37°C. The supernatant was then discarded, and 100 μL of dimethyl sulfoxide (DMSO) was added to dissolve the formazan crystals formed by viable cells. The absorbance of the solution was measured at 490 nm using a microplate reader. The cell viability was calculated by the following formula: Cell viability = (OD_490_ of Sample - OD_490_ of Blank)/(OD_490_ of Control–OD_490_ of Blank)*100%.

#### 2.5.1 Anti-inflammatory activity analysis via nitric oxide determination in RAW264.7 cells

RAW264.7 cells were seeded into 96-well plates at a density of 3╳10^5^ cells per well for 12 h before treatment. The cells were pretreated with different proportions of EM formulas (total 0.3 mg/mL) for 2 h. Subsequently, the cells were treated with LPS at a concentration of 1 μg/mL, along with the 76 prescriptions for 24 h. The supernatant was mixed with Griess reagent in a 96-well plate for 10 min. The absorbance of the solution was then measured at 530 nm using a microplate reader. The amount of NO produced was determined by comparing the absorbance to a sodium nitrite (NaNO_2_) standard curve. The anti-inflammatory activity inhibition ratio was calculated by the following formula: Anti-inflammatory activity inhibition ratio = (1-(OD_530_ of Sample - OD_530_ of Blank)/(OD_530_ of Model - OD_530_ of Blank))*100%.

#### 2.5.2 Anti-allergic activity analysis via degranulation assay in RBL-2H3 cells

The release of β-hexosaminidase from activated RBL-2H3 cells was measured as an indicator of degranulation as described elsewhere (Zeng et al., 2016). Briefly, RBL-2H3 cells were seeded into 96-well plates at a density of 3╳10^5^ cells per well for 12 h before treatment. The cells were pretreated with different proportions of EM formulas (total 1 mg/mL) for 2 h. Subsequently, the cells were stimulated with 50 nM PMA plus 1 μM A23187. After 5 h of stimulation, the supernatants (50 μL) were incubated with a substrate buffer (3.3 mM p-nitropheny-N-acetyl-β-D-glucosaminide, pH 4.5) in 96-well plates at 37°C for 1 h. The reaction was terminated using 100 μL stop solution (0.1 M sodium carbonate (Na_2_CO_3_)/sodium bicarbonate (NaHCO_3_), pH 10.2) and the absorbance was measured at 407 nm using a microplate reader. The anti-allergic activity inhibition ratio was calculated by the following formula: Anti-allergic activity inhibition ratio = (1-(OD_407_ of Sample - OD_407_ of Blank)/(OD_407_ of Model - OD_407_ of Blank))*100%.

### 2.6 Artificial neural network

The ANN model consisted of an input layer, a hidden layer and an output layer. The input layer had six nodes, representing the six types of Chinese medicinal materials. The outputs were the anti-inflammatory activity/anti-allergic activity inhibition ratio. The transfer function from the input layer to the hidden layer was Rectified Linear Unit (ReLU), while the transfer function from the hidden layer to the output layer was a sigmoid function. A DropOut strategy was implemented between the input layer and the hidden layer to improve the model’s performance. For training, validation, and testing the model, a total of 76 runs were conducted. Among these, 56 samples were used as the training set, 10 samples as the validation set, and the remaining 10 samples as the test set. To optimize the performance of the ANN model, various parameters were tested through trial and error analysis. These parameters included the number of nodes in the hidden layer (ranging from 5 to 60), the methods of weights and bias values updating (adam, sgd, rmsprop), the dropout ratio (ranging from 0.2 to 0.8), and the learning rate (0.001, 0.002, 0.005, 0.01). In the three-layer artificial neural network with m neurons in the hidden layer and n input variables, output $\hat{y}$ can be calculated as:

$$\hat{y}=f\left[\sum_{j=1}^{m}w_{j}\cdot g\left(\sum_{i=1}^{n}w_{ji}x_{i}+w_{j0}\right)+w_{0}\right]$$

Where *w*_j_: the weight that connects the *j*th neuron of hidden layer and neuron of the output layer, *w*_ji_: the weight that connects the *i*th input variable and *j*th neuron of the hidden layer, *x*_i_: the *i*th input variable, *w*_j0_: the bias of the *j*th neuron of the hidden layer, *w*_0_: bias related to the output neuron, *g*: the transfer functions for the hidden layer, and *f*: transfer functions for the output layer. The artificial neural network uses a group of random numbers to begin to train the network. The training process should be back propagation in such a way that the following function would be minimized:

$$E=\frac{1}{K}\sum_{k=1}^{K}{\left(y_{k}-{\hat{y}}_{k}\right)}^{2}$$

The methodology of “early stopping by cross-validation” was applied to prevent overfitting with the 10 validation samples.

The artificial neural network was built using TensorFlow 2.0 under Python 3.7. And TensorBoard was utilized to display the most suitable network parameters among the 21,840 candidate models.

### 2.7 Random forest regression (RF)

RF is an ensemble learning method that contains a large number of decision trees. In this study, the TreeBagger function in Matlab 2019b (Mathworks, Natick, MA, United States) with regression mode was used to grow the trees with the training data. Here, we operated the RF with the following parameters: ***ntree*** (number of trees to grow, or *n_estimators*) was set as 50; *NumPredictorsToSample* (number of predictor randomly sampled as candidates at each split, or *max_depth*) was set as 2; *MinLeafSize* (Minimum number of observations per tree leaf) was set as 5; *InBagFraction* (Fraction of observations that are randomly selected with replacement for each bootstrap replica) was set as 85% and data were sampled for each decision tree with replacement.

### 2.8 Support vector regression (SVR)

The principle of SVR is based on the structural risk minimization, which minimizes an upper bound of the generalization error. The basic idea of SVR is to map the input variables into a high dimensional feature space where they are linearly correlated with the output variable. To solve the curse of dimensionality, the kernel function (KF) is introduced and realize the nonlinear transformation. In this study, fitrsvm function in Matlab 2019b was used to realize the SVR algorithm. Bayesian optimization algorithm was used to screen the eligible parameters for fitrsvm. The parameters available for selection for *kernel function* included ‘Gaussian’ (Radial Basis Function (RBF) kernel), ‘linear SVR’, ‘Quadratic SVR’ and ‘Cubic SVR’. And another two crucial parameters, *BoxConstraint (penalty coeffient (C))* and *KernelScale* were explored within a log-scaled range spanning from 10^−3^ to 10^3^ to identify the most suitable configurations. The last parameter *epsilon (ɛ)* value was searched among positive values log-scaled in the range [0.001,100] × iqr (*Y*)/1.349, where *Y* is the response variable.

### 2.9 Non-dominated sorting genetic algorithm

In this research, the Non-dominated sorting genetic algorithm adopted a variant of NSGAII called VNSGAII. The VNSGAII algorithm is an evolutionary algorithm whose basic components are the chromosome structure, fitness functions, genetic operators and the VNSGAII process.

The objective of the research was to screen the best prescriptions consisting of 6 different proportions of medicinal materials. The candidate chromosome structure was designed as a double precision vector of n dimensions, where n was the number of medicinal materials in the prescription. In this study, n was equal to 6, represented as {x_1_, x_2_, x_3_, x_4_, x_5_, x_6_}. The constraints were that the sum of the vector should be 50 mg, and each element should be between 0 and 50 mg.

The goodness of each chromosome is quantified by the fitness functions. In this paper, the built ANN models were selected as the fitness functions. The predicted values of the anti-inflammatory activity/anti-allergic activity inhibition ratio, obtained from the ANN models, were used to evaluate the performance of each prescription.

Genetic operators provided the basic search mechanism of VNSGAII. Based on existing solutions in the population, new solutions are created with three basic types of operators: selection, crossover and mutation. The selection operator was performed before crossover and mutation, using the tournament algorithm with a size of 2 to choose parents for the next-generation based on their scaled values from the fitness functions. Crossover produced two new individuals based on two parents, while mutation modified one individual to produce a new solution. In this research, 80% of the population was used for crossover by the scatter function, and 5% of the population was used for mutation by uniform function.

The VNSGAII process consisted of the following phases:

Phase 1—Initialization: the initial population included 100 chromosomes constrained by prescription design experiment restrictions as described in section Simplex centroid mixture design.

Phase 2—Produce offspring:

a. Select 100 parents *P* ^*(t)*^ for the next-generation using the tournament algorithm on the current population.
b. Children are created from the selected parents by mutation and crossover to create 100 offspring *Q* ^*(t)*^ *.*

Phase 3—Produce parents:

a. Merge *P* ^*(t)*^ and *Q* ^*(t)*^ into one matrix *T* ^*(t)*^, to guarantee elitism (a mechanism, which ensures that all the best chromosomes are passed to the next-generation). Compute the rank and crowding distance for all individuals in *T* ^*(t)*^, and sort it into different fronts of descending domination rank according to the fast non-dominated sorting method. The theory of the fast non-dominated sorting method is illustrated as follows:

Function *F* = fast_non_dominated_sort (*T* ^*(t)*^):

  For each p in *T* ^*(t)*^:
   
$\text{Set }S_{p}=\varphi ,n_{p}=0$

   For each q in *T* ^*(t)*^:
    If (p dominates^#^ q) then

$S_{p}=S_{p}\cup \left\{q\right\}//\text{ add }q\text{ into the dominating solution set of }p\;\left(S_{p}\right)$

    Else if (q dominates p) then *n*_p_ = *n*_p_ + one.
   If (*n*_p_ = 0) then.
    *p*_rank_ = 1, *F*_1_ = *F*_1_ ∪ {*p*}//no solutions dominate *p*, *F*_1_ is the first rank in *T* ^*(t)*^
  end
  Set *i* = 1
  While *F*_*i*_≠φ//stop criterion
   
Set Q=φ

   For each p in *F*_*i*_:
    For each q in *S*_p_:
     
$n_{q}=n_{q}\;–\;1$

     
$\text{if }\left(n_{q}=0\right)\text{ then }//\text{ no solutions dominate }q\text{ in sets other than }\left\{F_{1}\;∼\;F_{i-1}\right\}$

      *q*_rank_ = *i* + 1, *Q* = *Q* ∪ {*q*}
   
Set i=i+1

   
$\text{Set }F_{i}=Q\;//\;F_{i}\text{ is the }i\text{th rank in }T^{\left(t\right)}$

End fast_non_dominated_sort.

# In the Pareto-optimal solutions z*, some solutions *x* ^(p)^ dominate other *x* ^(q)^, and it means: For ∀ *i*∈{1,2}, if *f*_*i*_ (*x* ^(p)^) ≤ *f*_*i*_ (*x* ^(q)^), then *x* ^(p)^ dominates *x* ^(q)^

b. Trim the *T* ^*(t)*^ to 100 individuals by retaining the appropriate number of individuals in each rank. A pre-defined geometric distribution sets the number of individuals in each front. The geometric distribution function can be illustrated as the formula: *n*_i_ = *r* × *n*_i-1_, where *n*_i_ is the maximum number of allowed individuals in the *i*th front and *r* (<1) is the reduction rate.

Phase 4—Stop criterion:

The stop criterion in Phase 4 is based on the maximum number of generations. If this criterion is not satisfied, the process goes back to Phase 2(b) and continues.

All the parameters of VNSGAII were listed in Table 1. The VNSGAII algorithm was implemented using MATLAB 2019b under Windows 10 operating system.

**TABLE 1:** VNSGAII parameters for the optimization.

VNSGAII	Parameters
Fitness function	ANN
Decision variables	6
Population size	100
Selection method	Tournament (size = 2)
Mutation functions	Adaptive feasible
Mutation rate	0.05
Crossover function	Intermediate, ratio = 1.0
Crossover fraction	0.8
Distance measure function	distancecrowding
Pareto front population fraction	0.35
Number of iterations	100–200

### 2.10 Study on drug combination rules based on the *Apriori* algorithm

In this research, a total of 113 prescriptions involving 230 botanical drugs for the treatment of AD were retrieved from the ‘Dictionary of Traditional Chinese Medicine Prescription’ to build the network of the drug combination (Peng, 1993). The *Apriori* algorithm was employed to explore the rule within the prescriptions. The support degree and confidence level are important parameters in the *Apriori* algorithm. The support degree represents the probability that two TCMs, A and B, appear together in a prescription. It indicates the frequency of the occurrence of the combination of A and B in the dataset. The confidence level, on the other hand, represents the probability of the appearance of TCM B in a prescription given that TCM A is already present in the same prescription. These two indicators reflect the drug compatibility tendency in statistics (Hu et al., 2023). In this research, a support threshold of above 5% and a confidence threshold of above 50% were set. MATLAB 2019b was used to implement the *Apriori* algorithm and Cytoscape 3.8 was used to visually process the network.

## 3 Results

### 3.1 Chemomics profiling of the EM prescription

**FIGURE 1:**
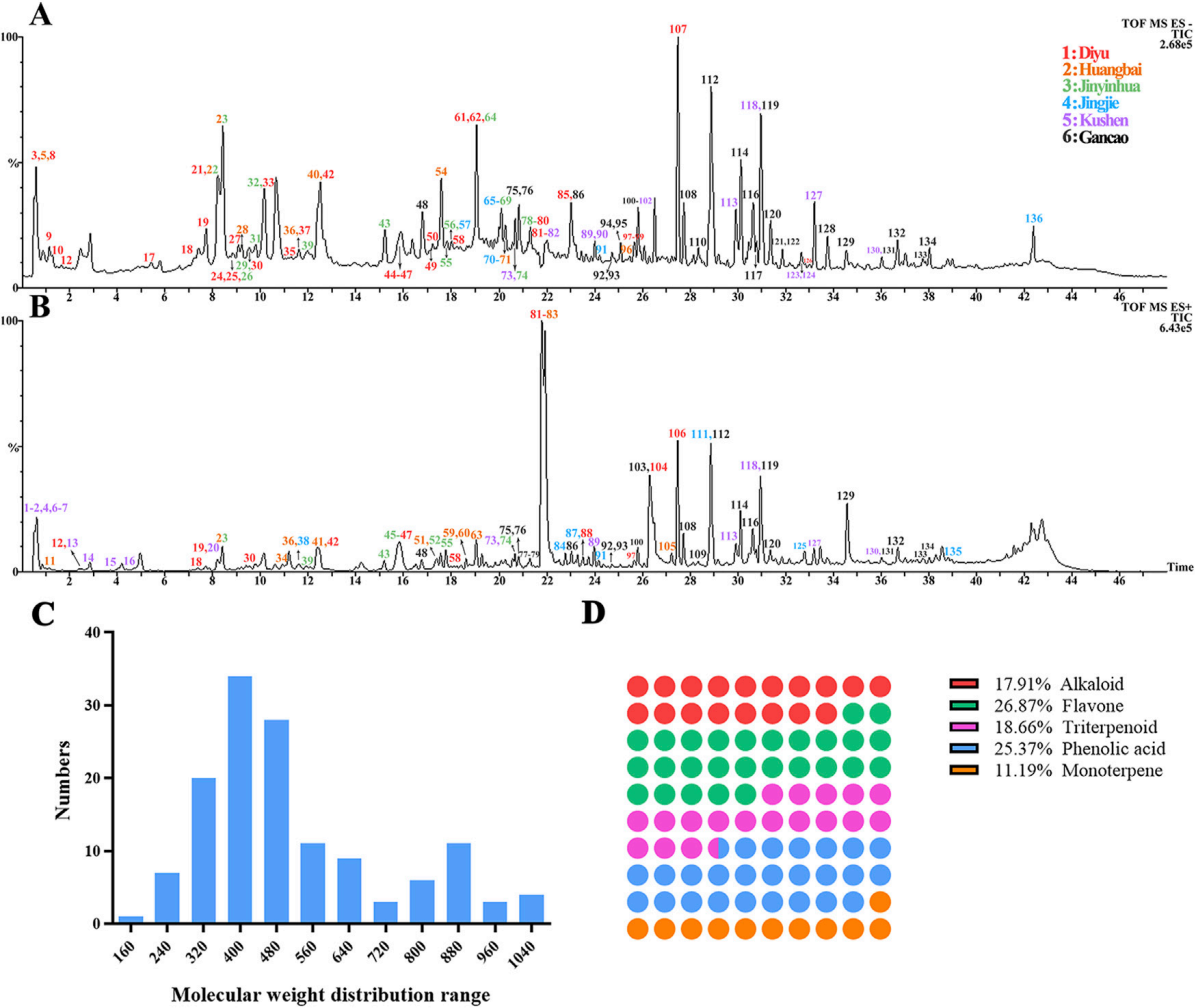
Chemomics profiling of EM prescription. TIC chromatogram in negative ESI mode **(A)** and positive ESI mode **(B)**; Molecular weight distribution for the identified compounds **(C)**; the main structure types in the EM prescription **(D)**.

To clarify the chemical composition of the EM prescription, the TIC chromatograms in both negative and positive modes of UPLC/Q-TOF/MS were obtained in Figures 1A,B. These chromatograms provided a visual representation of the compounds present in the EM prescription and their respective retention times. In total, 136 compounds were identified by comparing the retention times, m/z of the characteristic molecular and fragment ions. The detailed information about these identified compounds can be found in Supplementary Table S2. Among these compounds, alkaloids were found to be the main constituents derived from Huangbai and Kushen. The molecular weight distribution of these alkaloids ranged from 200 to 450 and they were the main pharmacodynamic compounds in the EM prescription (Figure 1C). The main structures of these alkaloids were categorized into different types, including quinolizidine-type, protoberberine-type, Apophis-type, and benzyl isoquinoline-type alkaloids. These structural classifications provided insights into the chemical diversity and complexity of the alkaloids present in the EM prescription (Figure 1D). Overall, the information presented in Figure 1 and Supplementary Table S2 shed light on the chemical composition of the EM prescription, specifically highlighting the presence of alkaloids derived from Huangbai and Kushen, their molecular weight distribution, and the main structural types of these alkaloids.

### 3.2 Back-propagation artificial neural network model

**FIGURE 2:**
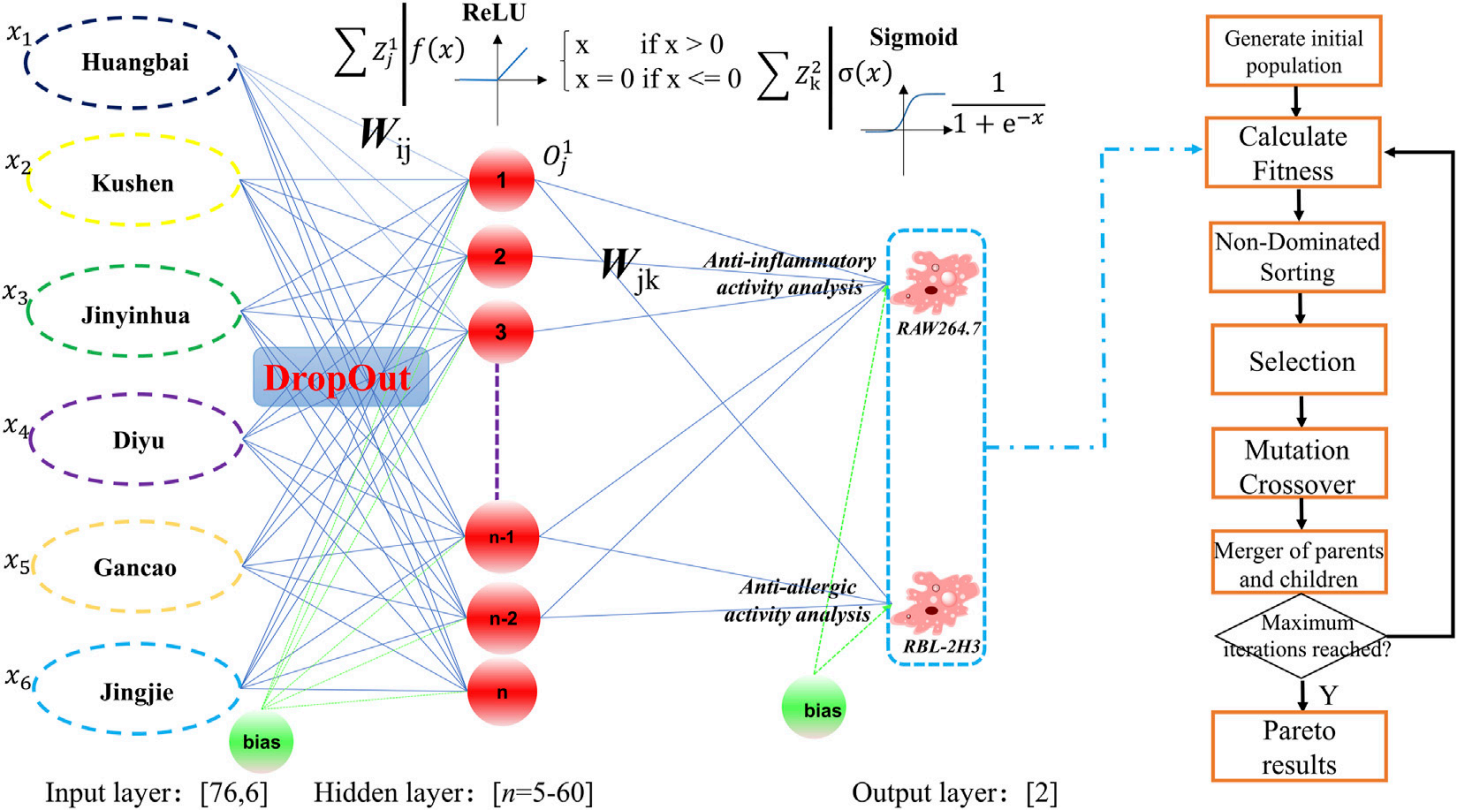
The schematic diagram of the proposed SCMD-ANN-VNSGAII methodology.

The schematic diagram of the proposed SCMD-ANN-VNSGAII methodology was presented in Figure 2. In the proposed methodology, BP-ANN was used to establish the relationship between the prescription ratio and the bioactivity. In the input layer, the data contained 76 rows and 6 columns where each row represented a prescription. The SCMD was used to design different prescriptions which were listed in Supplementary Table S1 and the total weight of each prescription was 50 mg. Their anti-inflammatory and anti-allergic bioactivities were acquired to build the ANN model. Briefly, RBL-2H3 cells were pretreated with different proportions of EM formulas (the concentration was 1 mg/mL), and RAW264.7 cells were pretreated with different proportions of EM formulas (the concentration was 0.3 mg/mL). No cytotoxic effects were detected at these concentrations (Supplementary Figure S1). Among these 76 runs, 56 samples were randomly selected as the calibration set to train the BP-ANN network, 10 samples were randomly selected as the validation set to monitor the training process to avoid overfitting and the remaining 10 samples were randomly selected as the test set. As for why to build the three layers of the BP-ANN Model (6-n-2), in our opinion, the input layer contained the six botanical drugs from the EM prescription. The hidden layer represented the main chemical compounds from the prescription, and the number of nodes was screened from 5 to 60 with the help of TensorBoard tools. The output layer contained the anti-inflammatory activity and the anti-allergic activity. In addition, to establish a robust artificial neural network model, the DropOut strategy and ReLU transfer function were introduced. The key idea of DropOut strategy is to randomly drop units (along with their connections) from the neural network during training. The dropout strategy was introduced during the input layer and hidden layer as shown in Figure 2. From the chemical sense, not all the components from the botanical drugs contributed to the bioactivities. It has been proven that the dropout strategy significantly reduces overfitting and gives major improvements over other regularization methods (Zhang and Xu, 2024). At the same times, the ReLU transfer function was executed after the DropOut strategy. The output of the ReLU neuron was a piecewise linear function which has been successfully applied to regression, classification and function approximation (Liang and Xu, 2021). The output of the ReLU function is max{0,*w* ^*T*^ *x* + *b*}, in which *w* and *b* are the weight and bias parameters, respectively. From the chemical sense, the nodes in the hidden layer were all positive which represented the concentration of the chemical components. Finally, the Sigmoid transfer function was used to simulate the half-inhibition rate of anti-inflammatory and ant-allergic activity whose values w ere both limited between 0% and 100%.

**FIGURE 3:**
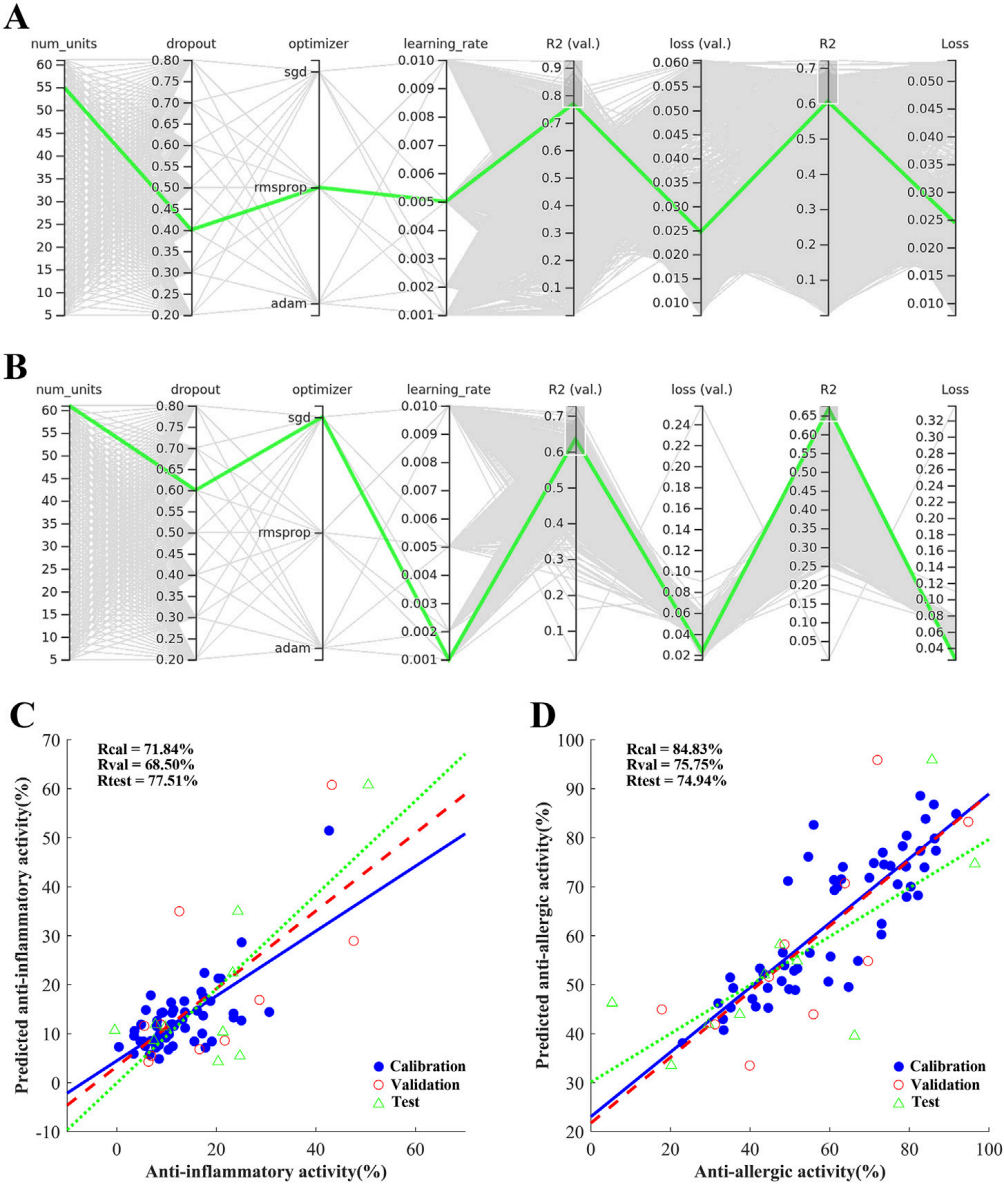
The TensorBoard view of different parameter combinations of BP-ANN for the anti-inflammatory activity **(A)**; the anti-allergic activity **(B)**; the scatter plot of model predicted vs. observed values of anti-inflammatory activity **(C)**; the anti-allergic activity **(D)**.

**FIGURE 4:**
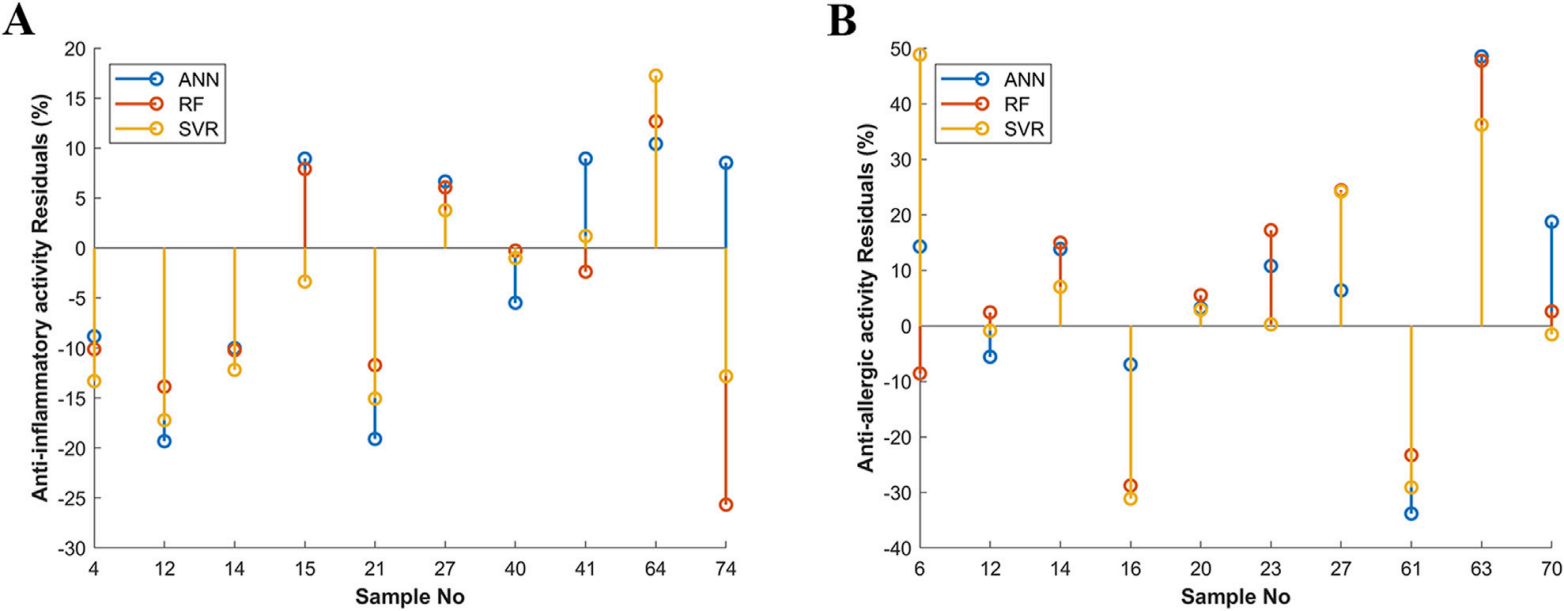
The residual plots of the anti-inflammatory activity **(A)** and the anti-allergic activity **(B)** in the test set.

Except for the BP-ANN framework, some parameters in the model were also optimized during training. These parameters included the dropout rate between the input layer and hidden layer, the number of nodes in the hidden layer, the gradient descent algorithms and the learning rate. A total of 21,840 parameter combinations were screened simultaneously, and the results were visualized using TensorBoard, as shown in Figures 3A,B. The green line in Figure 3A represented the most optimal parameter combination of the anti-inflammatory activity. Under the optimal parameters, the regression coefficient of the true value and predicted value of anti-inflammatory activity in the calibration was 71.84%. The validation set was used to avoid overfitting during training which also reached the value of 68.5%. The test set was used to evaluate the robustness of the model which also reached the value of 77.51% (Figure 3C). Furthermore, a good fitting effect was observed in the simulation of the anti-allergic activity, as shown in Figure 3D. At the same times, two another machine learning algorithms, SVR and RF, were benchmarked against the BP-ANN algorithm. After the hyperparameter tuning process for SVR, these two activities both selected the linear kernel function with the corresponding *kernel scale* values 0.0026 and 0.0012. The last parameter *epsilon (ɛ)* values were 0.9530 and 0.0322 respectively. For selection of the RF parameters for these two activities, we set *ntree* (number of trees to grow, or *n_estimators*) as 50 and *NumPredictorsToSample* (number of predictor randomly sampled as candidates at each split, or *max_depth*) as 2 based on our experience. As shown in Table 2 and Figure 4 and Supplementary Figures S2, 3), the BP-ANN algorithm exhibited lower RMSEP and higher *R*_test_ values compared to RF and SVR in the anti-inflammatory and anti-allergic activity. Nonetheless, it was worth noting that RF possessed the capability to assess variable importance, which could offer valuable insights into the relative significance of input features (Supplementary Figures S2C,D). Regarding the rationale for selecting a linear kernel over a Gaussian kernel in SVR, potential factors may be associated with the following two aspects: 1. Linear kernels demonstrate superior generalization performance in high-dimensional pharmacological datasets (n (56) ≫ p (6) scenario). 2. The reduced VC dimension of linear kernels (d = p + 1) effectively controls model complexity for TCM formula data.

**TABLE 2:** The comparison of the different machine learning algorithms in the NM stage of the ED-NM-MO trigeminy method.

Algorithm	Parameters		*R*_cal_		*RMSEP*			*R*_test_	
	Anti I^a^	Anti A^a^	Anti I	Anti A	Anti I	Anti A		Anti I	Anti A
BP-ANN	Structure:6–55–1	Structure:6–60–1	0.72	0.85	10.55	18.78		0.78	0.75
RF	Trees = 50, InBagFraction = 85% NumPredictorsToSample = 2		0.79	0.86	12.09	22.09		0.62	0.67
SVR	Kernal function = ‘Linear’		0.65	0.69	11.57	24.81		0.65	0.61

^a^ anti-inflammatory activity (Anti I); ant-allergic activity (Anti A).

Finally, these results collectively demonstrated the feasibility of the BP-ANN algorithm in the SCMD-ANN-VNSGAII methodology for building nonlinear relationships between bioactivities.

### 3.3 VNSGAII optimization process

**FIGURE 5:**
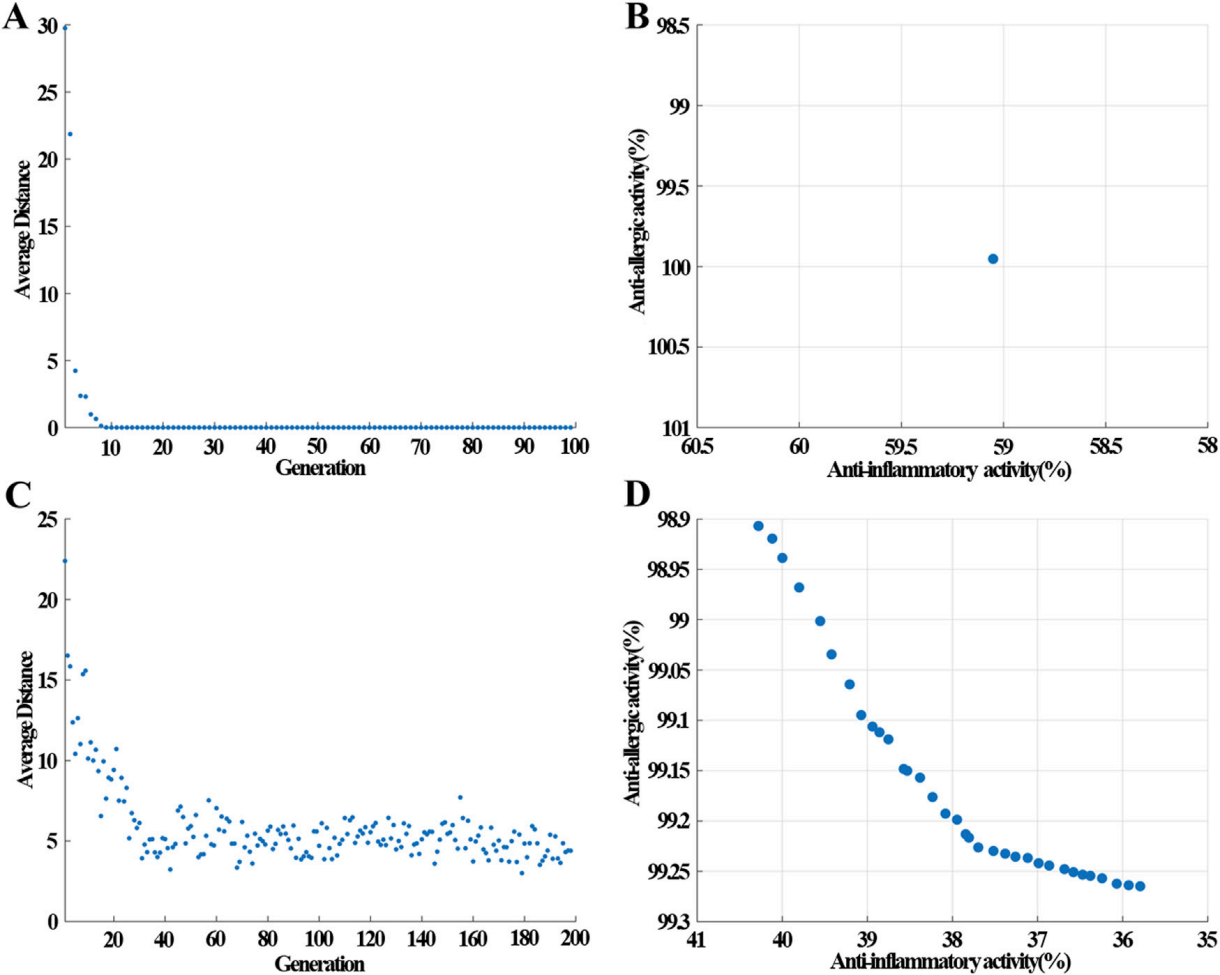
The change of average distance in each generation and the Pareto front obtained by VNSGAII at different restrictive conditions **(A,B)** without restrictions; **(C,D)** no single herb higher than 30 mg in the 50 mg prescription.

After establishing the BP-ANN model, VNSGAII shared the same seeds to begin the evolutionary algorithm for searching the best compatibility ratio of the EM prescription on anti-inflammatory and anti-allergic activity. This process was illustrated in Figure 2. Under the most suitable compatibility ratio, the bioactivity of the EM prescription would show excellent inhibition rates for both anti-inflammatory and anti-allergic activity. In Figure 5A, after 10 iterations, the VNSGAII algorithm successfully identified the optimal prescription, as evidenced by the gradual decrease and eventual convergence of the average distance between generations towards zero. This indicated that the algorithm effectively navigated the search space to locate the most suitable solution. The most suitable prescription consisted only of Huangbai herb,which resulted in an inhibition rate of 59.1% for anti-inflammatory activity and 100% for anti-allergic activity as shown in Figure 5B. This result suggested that Huangbai was the monarch medicine in the EM prescription and processed the function of removing damp heat, quenching fire, and counteracting toxicity (Ai et al., 2023). Some other similar prescriptions to cure AD almost included Huangbai (Zheng et al., 2021; Liu et al., 2022).

To identify the ministerial drug in the EM prescription, a limitation was set where the content of all botanical drugs should be less than 30 mg among the prescription with a total content of 50 mg. Under this restriction, the VNSGAII algorithm began to search for the best compatibility ratio of the EM prescription. After undergoing approximately 40 iterations, the VNSGAII algorithm converged upon the most suitable prescriptions, evident from the minimal fluctuations observed in the average distance metric across successive generations, as depicted in Figure 5C. The first rank of the Pareto plane after the VNSGAII algorithm was displayed in Figure 5D. Among these prescriptions, the anti-allergic inhibition rate ranged from 98.9% to 99.25% and the anti-inflammatory inhibition rate ranged from 35.8% to 40.2%. Although these bioactivities of the prescriptions with limitations were not as good as the single herb for the anti-inflammatory inhibition rate, and the inhibition rate decreased from 59.1% to 40.2%, it was important to note that the biological activity of TCM to cure AD was not only limited to the anti-allergic and anti-inflammatory functions. Some other activities, such as, T lymphocyte suppression activity and antibacterial activity may also be involved in the EM prescription. Restricting the prescription was beneficial for discovering potential active compatible ingredients.

### 3.4 Validation experiment

**FIGURE 6:**
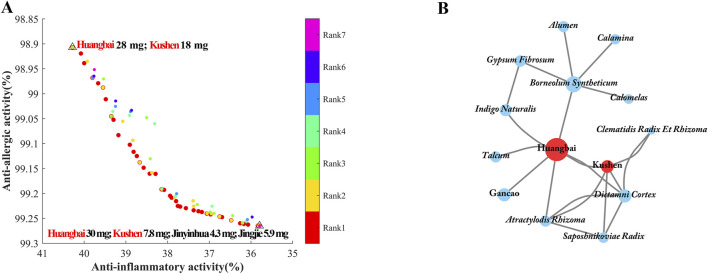
The Pareto front obtained by VNSGAII in the maximum number of generations at the restrictive condition of no single herb above 30 mg. **(A)** And the network of association rules of prescriptions to treat AD with the support above 5% and confidence above 50% **(B)**.

To verify the credibility of the SCMD-ANN-VNSGAII methodology, the 100 individuals obtained at the last iteration under the 30 mg content limitation were ranked into seven ranks with a geometric distribution, as shown in Figure 6A. The bioactivities of prescriptions in the first rank were better than those in other ranks. The best prescription with the highest anti-inflammatory activity consisted of 28 mg Huangbai and 18 mg Kushen in the total content of 50 mg prescription. The best prescription with the highest anti-allergic activity consisted of 30 mg Huangbai, 7.8 mg Kushen and some other small-weight of botanical drugs. These results reminded us that Huangbai and Kushen in the EM prescription were the most important botanical drugs to exert anti-AD activity.

**FIGURE 7:**
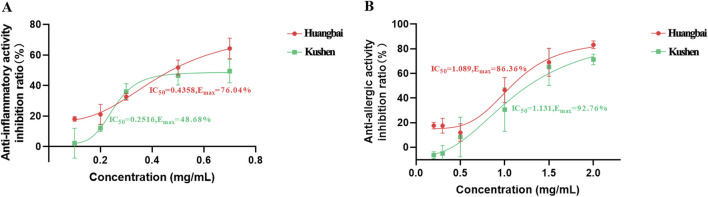
The comparison of Huangbai and Kushen in the anti-inflammatory activity **(A)** and anti-allergic activity **(B)** with the IC_50_ values and the maximum effect E_max_.

A total of 113 prescriptions involving 230 TCMs for the treatment of AD were retrieved from the ‘Dictionary of Traditional Chinese Medicine Prescription’ to build the network of the drug combination. The network of association rules of prescriptions to treat AD was shown in Figure 6B. The TCM interconnection network consisted of nodes and edges, where nodes represented different botanical drugs, the size of nodes represented the frequency of herb occurrence in all prescriptions. The edge connecting two botanical drugs represented that both of them appeared in the same prescription. Figure 6B showed that Huangbai and Kushen were often used in combination to treat AD, which was consistent with the results obtained from the SCMD-ANN-VNSGAII methodology. Furthermore, in Figure 7A, the IC_50_ values and maximum effect in the anti-inflammatory activity of Huangbai and Kushen were obtained. Huangbai showed a higher maximum effect (E_max_) than Kushen, but Kushen exhibited lower IC_50_ values. In Figure 7B, for the anti-allergic activity of Huangbai and Kushen, Huangbai showed higher Emax and lower IC50 values than Kushen. These results further confirmed that Huangbai was superior to Kushen in both anti-inflammatory and anti-allergic activity. Huangbai was used more frequently and more widely than Kushen in the medicinal frequency to cure AD.

## 4 Discussion

The combination of multiple botanical drugs in Traditional Chinese Medicine (TCM) is believed to elicit therapeutic effects through their interactions. However, the principle of combining specific herbal combinations is still unclear. In this study, the SCMD-ANN-VNSGAII methodology was proposed to search for the optimal compatibility ratio of an herbal combination known as EM. In fact, besides the TCM research, Western medicines for complex diseases also require the development of combination drug therapies. For example, combination therapies are used to treat drug resistance in cancer or infectious diseases, as well as to enhance the therapeutic effects of conditions like hypertension and diabetes (Güvenç Paltun et al., 2021). In Western medicines, two commonly used gold standards for quantifying synergy between drug combinations are Loewe additivity and Bliss independence. Loewe additivity is based on the assumption that the two inhibitors act through a similar mechanism while Bliss independence assumes independent mechanisms (Nguyen et al., 2024). However, these methods are only applicable to combinations of two drugs, and predicting synergies among multiple drugs remains a major challenge. In the present study of drug combinations, there are two main methods: the network-based approach and the construction of the machine learning model for prediction (Jafari et al., 2020; Wang et al., 2021). The SCMD-ANN-VNSGAII methodology proposed in this study falls under the machine learning model prediction method. Unlike other machine learning methods, this methodology does not require any information about chemical structures, signaling pathways, or genomic codes, as do the DeepSynergy, BestComboScore, ComboFM and Deep Tensor Factorization methods. Because all of these methods are based on Deep Learning methods and large-scale public databases, such as the NCI-60 human tumor cell line panel or research data from O’Neil et al., and they are not suitable to study the compatibility of CMF(Julkunen et al., 2020; Zhang et al., 2021). Due to the limited availability of publicly accessible biological activity data on TCM, more researchers tended to choose network-based methods rather than machine learning strategies to study CMF combinations (Wang et al., 2021; Niu et al., 2023).

In this study, we adopted the ‘ED-NM-MO trigeminy method’ which was a kind of rare machine learning strategy in CMF research. In the first part of the trigeminy method, we established a homemade database using SCMD, which included anti-inflammatory and anti-allergic biological activity data commonly used to evaluate the efficacy of AD treatment. Screening new drug combinations requires substantial efforts since considering all possible combinations between drugs is infeasible and expensive. Therefore, a compromise to obtain an acceptable model with the lowest number of experiments is needed. In the ED part, the optimal chemometric tool was employed to allocate the n experiments across the working space in a manner that maximized the informational yield for the given number of experiments. SCMD, which is a mixture design, was used to ensure that all mixtures in the matrix had the same final weight, with the differences between prescriptions being the proportion of medicinal materials. SCMD has been widely used in food and pharmaceutical research (Azhar and Munaim, 2021; M'Hir et al., 2023). Finally, the 76 rows × 6 columns homemade database was obtained with the corresponding anti-inflammatory and anti-allergic biological activity data. Then, Choosing the appropriate machine learning method was crucial. Considering the nonlinearity of biological activity, some linear algorithms such as multiple linear regression and partial least squares algorithms were excluded. At the same times, for the small-scale dataset, some Deep Learning algorithms including convolution neural networks, recurrent neural networks and deep belief networks were also excluded to avoid overfitting results, although the accuracy of Deep Learning algorithm, in all cases, is better than traditional machine learning methods. As for other traditional machine learning methods, such as support vector machine, ANN, Bayesian, decision tree and random forest methods, there is no clear leader in the biomedical problems (Tsigelny, 2019). Theoretically, a three-layer ANNs can approximate any function. Additionally, the BP-ANN model has been successfully applied in various medical applications. Notably, it has been successfully employed to forecast the blood concentration of monohydroxycarbazepine in epilepsy patients, enhancing treatment precision (Xu et al., 2024). Additionally, the model demonstrated remarkable accuracy in predicting the blood concentration of tacrolimus shortly after liver transplantation, leveraging mutation patterns or genomic profiles for more personalized and timely medical interventions (Du et al., 2024). Therefore, in this study, the ANN algorithm was adopted to build the relationship of the different formula ratios and the bioactivities. The schematic diagram of the proposed SCMD-ANN-VNSGAII methodology was illustrated in Figure 2. The detailed architecture of BP-ANN was determined through an exhaustive hyperparameter selection process, facilitated by the utilization of TensorBoard, ensuring optimal performance and efficiency. As the training algorithm, standard back-propagation was used. The optimal neural network architecture was searched for using the criteria of the highest regression coefficient *R*. The number of nodes in the hidden layer, the types of optimizer and the learning rate were trained for at least 100 epochs. It was found that a large number of units in the hidden layer, a smaller learning rate and the inclusion of the dropout regularization and ReLU transfer function were essential for achieving high-performing networks. One possible explanation for this is that the nodes in the hidden layer may represent the biological activity contribution of different compounds from different botanical drugs. These nodes in the hidden layer are similar to the defining of the pharmacophore. The Dropout regularization and ReLU transfer function help filter out non-pharmacologically active components. To assess the stability of the BP-ANN model, 10 samples were randomly selected as the test set. The regression coefficients *R* for the anti-inflammatory and anti-allergic activities of the test group reached 77.51% and 74.94%, respectively. These results are comparable to other artificial intelligence methods used in combination drug therapy, which also achieve a similar level of confidence in correct predictions between 0.7 and 0.9 (Tsigelny, 2019). At the same times, two another machine learning algorithms, SVR and RF, were benchmarked against the BP-ANN algorithm. The BP-ANN algorithm exhibited lower RMSEP and higher *R*_test_ values compared to RF and SVR in the anti-inflammatory and anti-allergic activity. After building the BP-ANN model, the focus shifted to finding the most ideal prescription for treating AD. In this study, two commonly used indicators to evaluate the efficacy of AD treatment, anti-inflammatory and anti-allergic biological activity were obtained. VNSGAII as a multi-objective optimization is an appropriate way to analyze the system when two or more objectives have equal importance and might conflict with each other. Compared to single-objective optimization, VNSGAII provides more objective results. VNSGAII is also an evolutionary algorithm, and each objective function is calculated separately and compared so that eventually all the non-dominated solutions are found and form the Pareto frontier. Accordingly, decision-makers can acquire the most suitable formula by considering all objectives and their trade-offs. In this study, it was rare that the Pareto plane only contained one point. This meant that in the treatment of AD, Huangbai not only had the strongest anti-inflammatory activity but also had the strongest anti-allergic activity. This result reminded us that the biological activities of TCM to cure AD were not only limited to the anti-allergic and anti-inflammatory functions. Some other activities, such as, T lymphocyte suppression activity and antibacterial activity may also be involved in the EM prescription. When the content of Huangbai in the total weight of 50 mg was limited to below 30 mg, Kushen, as the secondary important herb, exhibited supplemental anti-inflammatory and anti-allergic activities against Huangbai. The anti-inflammatory tests between Huangbai and Kushen also demonstrated that the E_max_ of Huangbai was greater than that of Kushen. There was no significant difference in the anti-allergic test between Huangbai and Kushen. Another validation experiment established a network consisting of 230 botanical drugs based on 113 prescriptions for treating AD through data mining in the ‘Dictionary of Traditional Chinese Medicine Prescription’. The herbal combination network indicated that Huangbai and Kushen were the core herb pairs for treating AD, and Huangbai was used more frequently than Kushen. It was worth noting that the RF algorithm provided the scores of feature importance, and Huangbai and Kushen were more import predictors for RF regression (Supplementary Figures S2C,D). These findings are consistent with the results obtained from the model used in this study. In further experiments, the SCMD-ANN-VNSGAII can be used to study the compatibility optimization at the level of homologous compounds among the Huangbai and Kushen, for example, the different alkaloid components among these two kinds of botanical drugs. Overall, these findings suggest that Huangbai and Kushen are promising herb pairs for treating AD, and further research can be conducted to optimize their compatibility and explore the specific compounds responsible for their therapeutic effects.

## 5 Conclusion

This research provided a methodology to study the compatibility optimization strategy of the EM prescription which included six kinds of Chinese medicinal materials. The methodology involved using SCMD to generate a dataset of formulas with a constant total amount of 50 mg. A three-layer BP-ANN algorithm was then used to predict the nonlinear bioactivity relationship, with the introduction of dropout strategy and ReLU transfer function to improve the model’s performance. Finally, the VNSGAII algorithm, as one of the most effective multi-objective genetic algorithms, which could supply many Pareto optimal solutions for researchers to help them “have their cake and eat it”, was used to search for the best compatibility ratio of the EM prescription on both anti-inflammatory and anti-allergic activity. As a result, Huangbai and Kushen were the main bioactive botanical drugs among these six botanical drugs which was in accordance with the drug combination rule based on the *Apriori* algorithm. In addition, the IC_50_ and E_max_ values of these two botanical drugs were acquired, and Huangbai was superior to Kushen in both the anti-inflammatory and anti-allergic activity. In conclusion, the proposed SCMD-ANN-VNSGAII as a powerful methodology has been proven to be successful in studying the compatibility of crude extracts, and this methodology may pave the way for future compatibility optimization of homologous compounds or specific component mixtures.

## Data Availability

The raw data supporting the conclusions of this article will be made available by the authors, without undue reservation.

## Abbreviations

CMF, Chinese medicine formula; TCM, traditional Chinese medicine; EM, Eczema mixture; UPLC-Q/TOF, Ultra-performance liquid chromatography-quadrupole/time-of-flight; SCMD, simplex centroid mixture design; BP-ANN, Back-Propagation artificial neural network; VNSGAII, a variant of non-dominated sorting genetic algorithm II; MTT, 3-(4,5-dimethylthiazol-2-yl)-2,5-diphenyltetrazolium bromide; DMSO, dimethyl sulfoxide; DXM, Dexamethasone; Huangbai, *Phellodendri Chinensis* Cortex; Diyu, *Sanguisorbae* Radix; Kushen, *Sophorae Flavescentis* Radix; Gancao, *Glycyrrhizae Radix Et Rhizoma*; Jinyinhua, *Honeysuckle* Buds; Jingjie, *Schizonepetae* Herba; AD, atopic dermatitis; NO, nitric oxide; LPS, lipopolysaccharide; PMA, Phorbol 12-myristate 13-acetate; ED-NM-MO trigeminy method, Experimental Design - Nonlinear Modeling - Multi-objective Optimization trigeminy method; ReLU, rectified linear unit; DMEM, Dulbecco’s modified Eagle’s medium; FBS, Fetal bovine serum.
