# Supplementary material for: Compatibility optimization of the traditional Chinese medicines ‘Eczema mixture’ based on back-propagation artificial neural network and non-dominated sorting genetic algorithm
Source: Front Pharmacol. 2025 May 2;16:1593783. doi: 10.3389/fphar.2025.1593783 (PMC12082046; doi:10.3389/fphar.2025.1593783)
Supplement: Supplementary file 1 [file Table1.docx]

Table S1 Proportion of components in ‘Eczema mixture’ formulas

| Run | Huangbai | Diyu | Kushen | Gancao | Jinyinhua | Jingjie |
| --- | --- | --- | --- | --- | --- | --- |
| 1 | 0 | 12.5 | 12.5 | 12.5 | 0 | 12.5 |
| 2 | 12.5 | 0 | 0 | 12.5 | 12.5 | 12.5 |
| 3 | 10 | 10 | 10 | 0 | 10 | 10 |
| 4 | 0 | 0 | 16.67 | 0 | 16.67 | 16.67 |
| 5 | 4.17 | 29.17 | 4.17 | 4.17 | 4.17 | 4.17 |
| 6 | 50 | 0 | 0 | 0 | 0 | 0 |
| 7 | 10 | 0 | 10 | 10 | 10 | 10 |
| 8 | 12.5 | 0 | 12.5 | 12.5 | 0 | 12.5 |
| 9 | 12.5 | 12.5 | 12.5 | 12.5 | 0 | 0 |
| 10 | 16.67 | 16.67 | 0 | 0 | 16.67 | 0 |
| 11 | 4.17 | 4.17 | 29.17 | 4.17 | 4.17 | 4.17 |
| 12 | 0 | 0 | 0 | 0 | 0 | 50 |
| 13 | 0 | 0 | 0 | 50 | 0 | 0 |
| 14 | 0 | 0 | 0 | 0 | 50 | 0 |
| 15 | 0 | 0 | 50 | 0 | 0 | 0 |
| 16 | 12.5 | 12.5 | 12.5 | 0 | 0 | 12.5 |
| 17 | 10 | 10 | 0 | 10 | 10 | 10 |
| 18 | 0 | 12.5 | 12.5 | 12.5 | 12.5 | 0 |
| 19 | 0 | 0 | 0 | 0 | 50 | 0 |
| 20 | 0 | 12.5 | 0 | 12.5 | 12.5 | 12.5 |
| 21 | 0 | 50 | 0 | 0 | 0 | 0 |
| 22 | 0 | 0 | 0 | 0 | 0 | 50 |
| 23 | 0 | 0 | 50 | 0 | 0 | 0 |
| 24 | 12.5 | 0 | 12.5 | 12.5 | 12.5 | 0 |
| 25 | 29.17 | 4.17 | 4.17 | 4.17 | 4.17 | 4.17 |
| 26 | 0 | 10 | 10 | 10 | 10 | 10 |
| 27 | 0 | 0 | 0 | 50 | 0 | 0 |
| 28 | 8.33 | 8.33 | 8.33 | 8.33 | 8.33 | 8.33 |
| 29 | 0 | 0 | 0 | 16.67 | 16.67 | 16.67 |
| 30 | 12.5 | 0 | 12.5 | 0 | 12.5 | 12.5 |
| 31 | 0 | 0 | 16.67 | 16.67 | 0 | 16.67 |
| 32 | 0 | 0 | 12.5 | 12.5 | 12.5 | 12.5 |
| 33 | 16.67 | 0 | 0 | 0 | 16.67 | 16.67 |
| 34 | 25 | 0 | 0 | 0 | 0 | 25 |
| 35 | 25 | 0 | 0 | 0 | 25 | 0 |
| 36 | 0 | 16.67 | 16.67 | 0 | 0 | 16.67 |
| 37 | 0 | 25 | 0 | 25 | 0 | 0 |
| 38 | 0 | 25 | 25 | 0 | 0 | 0 |
| 39 | 16.67 | 0 | 16.67 | 16.67 | 0 | 0 |
| 40 | 0 | 25 | 0 | 0 | 25 | 0 |
| 41 | 25 | 0 | 0 | 25 | 0 | 0 |
| 42 | 0 | 16.67 | 0 | 0 | 16.67 | 16.67 |
| 43 | 0 | 0 | 25 | 0 | 25 | 0 |
| 44 | 25 | 25 | 0 | 0 | 0 | 0 |
| 45 | 16.67 | 0 | 16.67 | 0 | 0 | 16.67 |
| 46 | 0 | 16.67 | 0 | 16.67 | 16.67 | 0 |
| 47 | 0 | 0 | 16.67 | 16.67 | 16.67 | 0 |
| 48 | 16.67 | 16.67 | 16.67 | 0 | 0 | 0 |
| 49 | 16.67 | 16.67 | 0 | 16.67 | 0 | 0 |
| 50 | 0 | 25 | 0 | 0 | 0 | 25 |
| 51 | 0 | 0 | 0 | 25 | 0 | 25 |
| 52 | 10 | 10 | 10 | 10 | 10 | 0 |
| 53 | 4.17 | 4.17 | 4.17 | 29.17 | 4.17 | 4.17 |
| 54 | 12.5 | 12.5 | 0 | 0 | 12.5 | 12.5 |
| 55 | 0 | 0 | 0 | 25 | 25 | 0 |
| 56 | 16.67 | 0 | 16.67 | 0 | 16.67 | 0 |
| 57 | 4.17 | 4.17 | 4.17 | 4.17 | 4.17 | 29.17 |
| 58 | 10 | 10 | 10 | 10 | 0 | 10 |
| 59 | 16.67 | 0 | 0 | 16.67 | 0 | 16.67 |
| 60 | 12.5 | 12.5 | 0 | 12.5 | 0 | 12.5 |
| 61 | 0 | 0 | 25 | 25 | 0 | 0 |
| 62 | 0 | 16.67 | 0 | 16.67 | 0 | 16.67 |
| 63 | 0 | 16.67 | 16.67 | 0 | 16.67 | 0 |
| 64 | 16.67 | 16.67 | 0 | 0 | 0 | 16.67 |
| 65 | 12.5 | 12.5 | 0 | 12.5 | 12.5 | 0 |
| 66 | 16.67 | 0 | 0 | 16.67 | 16.67 | 0 |
| 67 | 25 | 0 | 25 | 0 | 0 | 0 |
| 68 | 0 | 0 | 0 | 0 | 25 | 25 |
| 69 | 0 | 16.67 | 16.67 | 16.67 | 0 | 0 |
| 70 | 0 | 50 | 0 | 0 | 0 | 0 |
| 71 | 0 | 12.5 | 12.5 | 0 | 12.5 | 12.5 |
| 72 | 12.5 | 12.5 | 12.5 | 0 | 12.5 | 0 |
| 73 | 4.17 | 4.17 | 4.17 | 4.17 | 29.17 | 4.17 |
| 74 | 50 | 0 | 0 | 0 | 0 | 0 |
| 75 | 0 | 0 | 25 | 0 | 0 | 25 |
| 76 | 0 | 0 | 25 | 0 | 0 | 25 |

Table S2 MS/MS data from ESI-MS and identification of the ‘Eczema mixture’ formula (1:1:1:1:1:1)

| No | tR | MS[M+H]+ | MS/MS | MS[M-H]- | MS/MS | Formula | Compound | From |
| --- | --- | --- | --- | --- | --- | --- | --- | --- |
| 1 | 0.61 | 205.1302 | 191,148,136,114,96 |  |  | C12H16N2O | N-methylcytisine | Kushen |
| 2 | 0.61 | 191.1174 | 148,136,114,96 |  |  | C11H14N2O | Cytisine | Kushen |
| 3 | 0.61 |  |  | 331.0695 | 301,211,169 | C13H16O10 | 1-gallic acid acylglucose | Diyu |
| 4 | 0.62 | 249.1966 | 205,191,150,136,96 |  |  | C15H24N2O | Sophoridine | Kushen |
| 5 | 0.62 |  |  | 341.1074 | 191 | C16H22O8 | Coniferin | HuangBai |
| 6 | 0.64 | 265.1891 | 247,203,150,136 |  |  | C15H24N2O2 | Sophoranol | Kushen |
| 7 | 0.65 | 263.1757 | 245,203,177,150,136,96 |  |  | C15H22N2O2 | 9a-Hydroxysophocarpine | Kushen |
| 8 | 0.85 |  |  | 191.0207 | 111 | C6H8O7 | Citric Acid | Diyu |
| 9 | 1.16 |  |  | 331.0695 | 301,211,169 | C13H16O10 | 1-gallic acid acylglucose | Diyu |
| 10 | 1.30 |  |  | 169.0160 |  | C7H6O5 | Gallic acid | Diyu |
| 11 | 1.37 | 180.1373 | 121,103,93,77 |  |  | C11H18NO^+^ | Candicine | Huangbai |
| 12 | 2.62 | 307.0791 | 139 | 305.0669 | 167,137,125 | C15H14O7 | (-)-gallocatechin | Diyu |
| 13 | 2.85 | 249.1966 | 175,148,107 |  |  | C15H24N2O | Matrine | Kushen |
| 14 | 3.25 | 247.1807 | 179,150,136,96 |  |  | C15H22N2O | Sophocarpine | Kushen |
| 15 | 3.98 | 263.1757 | 245,203,177,150,136,98 |  |  | C15H22N2O2 | Oxysophocarpine | Kushen |
| 16 | 4.69 | 265.1891 | 247,205,148,136 |  |  | C15H24N2O2 | Oxymatrine | Kushen |
| 17 | 5.81 |  |  | 345.0832 | 169,124 | C14H18O10 | methyl-6-O-galloyl-β-D-glucopyranoside | Diyu |
| 18 | 7.21 | 579.1526 | 409,287,247,139 | 577.1388 | 425,407,289,125 | C30H26O12 | Procyanidin B3 | Diyu |
| 19 | 7.72 | 291.0842 | 139,123 | 289.0747 | 137,123,109 | C15H14O6 | Cianidanol | Diyu |
| 20 | 7.83 | 265.1891 | 150 |  |  | C15H24N2O2 | Lamprolobine | Kushen |
| 21 | 8.06 | 867.2074 | 579,427,409,289,247 | 865.2031 | 695,577,483,301,287 | C45H38O18 | procyanidin C2 | Diyu |
| 22 | 8.17 | 377.0851 | 163 | 353.0884 | 191 | C16H18O9 | 5-O-caffeoyquinic acid | Jinyinhua/ Huangbai |
| 23 | 8.39 | 377.0851 | 163 | 353.0884 | 191 | C16H18O9 | 3-O-caffeoyquinic acid | Jinyinhua / Huangbai |
| 24 | 8.61 |  |  | 483.0801 |  | C20H20O14 | 2-gallic acid acylglucose | Diyu |
| 25 | 8.81 |  |  | 483.0801 |  | C20H20O14 | 2-gallic acid acylglucose | Diyu |
| 26 | 8.92 |  |  | 375.1302 | 213,191,174,169,113 | C16H24O10 | 8-epi-Loganin acid | Jinyinhua |
| 27 | 9.06 |  |  | 633.0797 | 301,197 | C27H22O18 | Sanguiin H4 | Diyu |
| 28 | 9.22 |  |  | 367.1048 | 193,134 | C17H20O9 | 3-O-feruloylquinic acid | Huangbai |
| 29 | 9.53 |  |  | 375.1302 | 341 | C16H24O10 | Loganin acid | Jinyinhua |
| 30 | 9.57 | 293.0265 | 286,247,219,191,163 | 291.0157 |  | C13H8O8 | Brevifolincarboxylic acid | Diyu |
| 31 | 9.82 |  |  | 389.1090 | 121 | C17H26O10 | Loganin | Jinyinhua |
| 32 | 10.18 |  |  | 373.1158 | 193 | C16H22O10 | Swertiamarin | Jinyinhua |
| 33 | 10.27 |  |  | 277.0028 | 197,182,167,123 | C9H10O8S | Methoxygallate-5-O-sulfate | Diyu |
| 34 | 10.97 | 342.1724 | 192,177 |  |  | C20H24NO4^+^ | Phellodendrine | Huangbai |
| 35 | 11.15 |  |  | 635.0840 | 465,313,169 | C27H24O18 | 3-gallic acid acylglucose | Diyu |
| 36 | 11.52 | 448.1985 | 349,286,255 | 446.1841 | 284,193,176,134 | C23H29NO8 | N-methylhigenamine-7-O-β-D-glucopyranoside | Huangbai |
| 37 | 11.59 |  |  | 635.0840 | 465,313,169 | C27H24O18 | 3-gallic acid acylglucose | Diyu |
| 38 | 11.63 | 349.1843 | 305 |  |  | C16H28O8 | Schizonepetoside E | Jingjie |
| 39 | 12.11 | 381.1169 | 297,197,127 | 403.1258 | 341 | C16H22O9 | Sweroside | Jinyinhua |
| 40 | 12.30 | 342.1686 | 297,265,237,177 |  |  | C20H24NO4^+^ | Magnoflorine | Huangbai |
| 41 | 12.49 |  |  | 367.1048 | 191,173,134,93 | C17H20O9 | 5-O-feruloylquinic acid | Huangbai |
| 42 | 12.64 | 731.1614 | 409,271,153 | 729.1417 | 577,407,289 | C37H30O16 | 3-O-galloylprocyanidin B-3 | Diyu |
| 43 | 15.21 | 427.1176 | 323,165,151 | 403.1258 | 371,121 | C17H24O11 | Secoxyloganin | Jinyinhua |
| 44 | 15.62 |  |  | 291.0157 | 211 | C10H12O8S | Methyl 3,4-Di-methoxybenzoate-5-O-sulfate | Diyu |
| 45 | 15.81 | 411.1281 | 327,246,165 | 433.1331 | 225,155 | C17H24O10 | 7-epi-vogeloside/vogeloside | Jinyinhua |
| 46 | 15.83 | 435.0583 |  | 433.0351 | 301 | C20H18O11 | Guaijaverin | Diyu |
| 47 | 15.88 | 303.0127 |  | 300.9957 |  | C14H6O8 | Ellagic acid | Diyu |
| 48 | 16.76 | 441.1165 | 257,137 | 417.1230 | 255,135,119 | C21H22O9 | Liquiritin | Gancao |
| 49 | 17.09 |  |  | 787.0926 |  | C34H28O22 | 2,3,4,6-Tetra-O-galloyl-β-D-glucose | Diyu |
| 50 | 17.20 |  |  | 787.0926 | 617,465,301,169 | C34H28O22 | 1,2,3,6-Tetra-O-galloyl-β-D-glucose | Diyu |
| 51 | 17.31 | 356.1863 | 311,296,279,264,248 |  |  | C21H26NO4^+^ | Menisperine | Huangbai |
| 52 | 17.43 | 611.1601 | 341,303 | 609.1539 | 300 | C27H30O16 | Rutin | Jinyinhua |
| 53 | 17.58 |  |  | 549.1624 | 255,135 | C26H30O13 | licraside | Gancao |
| 54 | 17.59 | 328.1917 | 283,121 |  |  | C19H22NO4^+^ | Tetrahydroreticuline | Huangbai |
| 55 | 17.77 | 538.2308 | 481,376,358 | 536.2210 | 312,164 | C26H35NO11 | L-Phenylalaninosecologanin | Jinyinhua |
| 56 | 17.99 | 449.1078 | 287 | 447.0966 | 285 | C21H20O11 | Luteolin-7-O-β-D-galactoside | Jinyinhua |
| 57 | 18.05 | 449.1078 | 305,287,130 | 447.0922 | 174,146 | C21H20O11 | Quercitrin | Jingjie |
| 58 | 18.25 | 443.0980 |  | 441.0771 |  | C22H18O10 | (-)-epicatechin gallate | Diyu |
| 59 | 18.67 | 324.1198 | 308,280,266 |  |  | C19H18NO4^+^ | Demethyleneberberine | Huangbai |
| 60 | 18.79 | 352.1164 | 336,308,294 |  |  | C20H17NO5 | Oxyberberine | Huangbai |
| 61 | 18.93 |  |  | 939.1035 | 315,301,300,169 | C21H10O13 | sanguisorbic acid dilactone | Diyu |
| 62 | 18.93 |  |  | 939.1035 | 769,301,169,125 | C41H32O26 | 1,2,3,4,6-Penta-O-galloyl-β-D-glucose | Diyu |
| 63 | 18.94 | 356.1863 | 192,177 |  |  | C21H25NO4 | Tetrahydropalmatine | Huangbai |
| 64 | 19.05 | 517.1436 | 163 | 515.1204 | 353,191,179,135 | C25H24O12 | 3,5-O-dicaffeoylquinic acid | Jinyinhua |
| 65 | 19.99 |  |  | 359.0770 | 197,179,161 | C18H16O8 | Rosmarinic acid | Jingjie |
| 66 | 20.00 |  |  | 408.9806 | 329,314,298,270,169 | C16H10O11S | 3,3'-Di-methylellagic acid-4-O-sulphate | Diyu |
| 67 | 20.00 | 803.1301 |  | 801.1039 | 649,408,298,270,169 | C35H30O22 | Methyl 2,3,4,6-tetra-O-galloyl-β-D-glucopyranoside | Diyu |
| 68 | 20.09 | 322.1062 | 307,279 |  |  | C19H15NO4 | Berberrubine | Huangbai |
| 69 | 20.11 | 517.1436 | 163 | 515.1204 | 353,191,179,135 | C25H24O12 | 3,4-O-dicaffeoylquinic acid | Jinyinhua |
| 70 | 20.27 | 611.2005 | 303,287,177,153 | 609.1843 | 301,285 | C28H34O15 | Hesperidin | Jingjie |
| 71 | 20.27 | 338.1385 | 322,308,294,280 |  |  | C20H20NO4^+^ | Columbamine | Huangbai |
| 72 | 20.45 | 338.1385 | 322,308,294,280 |  |  | C20H20NO4^+^ | Jatrorrhizine | Huangbai |
| 73 | 20.57 | 563.1782 | 269 | 561.1620 | 267,252,223,195,132 | C27H30O13 | Kushenol O | Kushen |
| 74 | 20.68 | 781.2562 |  | 757.2545 | 595,525,493,179 | C34H46O19 | (E)-Aldosecologanin | Jinyinhua |
| 75 | 20.83 | 551.1736 | 257,137 | 549.1624 | 255,135 | C26H30O13 | Liquiritin apioside | Gancao |
| 76 | 20.92 | 419.1328 | 295,257,147,137 | 417.1188 | 255,148,119 | C21H22O9 | Neoliquiritin | Gancao |
| 77 | 21.21 | 551.1736 | 269,257,147,137 | 549.1624 | 429,255,135 | C26H30O13 | Isoliquiritin apioside | Gancao |
| 78 | 21.31 | 781.2562 |  | 757.2545 | 595,525,493,179 | C34H46O19 | (Z)-Aldosecologanin | Jinyinhua |
| 79 | 21.34 | 419.1370 |  | 417.1188 |  | C21H22O9 | Neoisoliquiritin | Gancao |
| 80 | 21.65 |  |  | 408.9889 | 329,314,298,270 | C16H10O11S | 3,4'-Di-methylellagic acid-4-O-sulphate | Diyu |
| 81 | 22.16 | 463.0858 | 369,331,316 | 461.0750 | 329,314,299,271 | C21H18O12 | 3-4'-di-O-methyl ellagic acid-4'-O-β-D-xylopyranoside | Diyu |
| 82 | 22.17 | 285.0717 | 213,197,137,89 | 283.0634 | 268,239,211,175,147 | C16H12O5 | 3‘,7-Dihydroxy-4’-methoxy isoflavone | Kushen |
| 83 | 22.17 | 336.1268 | 336,320,308,292,278 |  |  | C21H22NO4^+^ | Berberine | Huangbai |
| 84 | 22.83 | 167.1059 | 149,130 |  |  | C10H14O2 | Z,E-nepetalactone | Jingjie |
| 85 | 22.86 |  |  | 423.0067 | 343,328,313,297,269 | C17H12O11S | 3,3',4'-Tri-methylellagic acid-4-O-sulphate | Diyu |
| 86 | 23.22 | 269.0813 |  | 267.0677 | 252,223 | C15H8O5 | Coumestrol | Gancao |
| 87 | 23.64 | 167.1059 | 149,130 |  |  | C10H14O2 | E,Z-nepetalactone | Jingjie |
| 88 | 23.64 | 471.2194 | 335,291,231 | 493.2293 |  | C21H36O10 | geranyl-1-O-β-D-xylopyranosyl-(1→6)-β-D-glucopyranoside | Diyu |
| 89 | 23.99 | 533.1307 | 285,151,123 | 1063.2343 | 283 | C25H24O13 | Trifolirhizin 6'-O-malonate | Kushen |
| 90 | 23.99 |  |  | 487.1271 | 283,255 | C24H24O11 | Trifolirhizin 6'-O-monoacertate | Kushen |
| 91 | 24.26 | 303.0839 | 130 | 301.0702 | 174,146 | C16H14O6 | Hesperetin | Jingjie |
| 92 | 24.74 | 1001.4644 | 825,649,631,487,469 | 999.4490 |  | C48H72O22 | 24-hydroxyl-Licorice-saponine A3 | Gancao |
| 93 | 24.83 | 897.4074 | 545,527 | 895.3989 |  | C44H64O19 | Uralsaponin F | Gancao |
| 94 | 25.09 | 855.4012 | 503,485,467 | 853.3929 |  | C42H62O18 | 22-hydroxyl-Licorice-saponine G2 | Gancao |
| 95 | 25.36 | 257.0799 |  | 255.0673 |  | C15H12O4 | liquiritigenin | Gancao |
| 96 | 25.51 |  |  | 487.1994 | 457,337,304,174 | C26H32O9 | Obacunoic acid | Huangbai |
| 97 | 25.64 | 345.0598 | 330,313 | 343.0450 | 328,313,298,270 | C17H12O8 | 3,3',4'-Tri-methylellagic acid | Diyu |
| 98 | 25.66 |  |  | 1025.4653 |  | C50H74O22 | 22-acetoxyl-rhaoglycyrrhizin | Gancao |
| 99 | 25.70 |  |  | 695.4012 | 487,343,312,297,269 | C36H56O13 | 2α,3,19-trihydroxyurs-12-en-24,28-dioic acid 28-β-D-glucopyranosyl ester | Diyu |
| 100 | 25.83 | 985.4667 | 809,615,471,453 | 983.4453 |  | C48H72O21 | Licorice saponine A3 | Gancao |
| 101 | 25.88 |  |  | 695.4012 | 603,487 | C36H56O13 | 2α,3,19-trihydroxyurs-12-en-24,28-dioic acid 28-β-D-glucopyranosyl ester | Diyu |
| 102 | 25.97 | 269.0813 | 253,237,226,213,197,118 | 267.0677 | 252,223,195,175 | C16H12O4 | Formononetin | Kushen |
| 103 | 26.09 | 881.4112 | 705,529,511,451 | 879.3987 | 351 | C44H64O18 | 22-acetoxyl-glycyrrhizin | Gancao |
| 104 | 26.29 |  |  | 695.4012 | 487 | C36H56O13 | 2α,3,19-trihydroxyurs-12-en-24,28-dioic acid 28-β-D-glucopyranosyl ester | Diyu |
| 105 | 27.24 | 471.2017 | 465,425,161 | 469.1904 | 229,174,146 | C26H30O8 | Limonin | Huangbai |
| 106 | 27.46 | 455.3543 | 437,201,191 |  |  | C30H46O3 | Sanguisorbigenin | Diyu |
| 107 | 27.51 |  |  | 811.4516 | 603 | C41H66O13 | Ziyu glycoside I | Diyu |
| 108 | 27.74 | 839.4065 | 487,469,451 | 837.4012 | 351 | C42H62O17 | Licorice saponin G2 | Gancao |
| 109 | 28.14 | 839.4065 | 487,469,451 | 837.3893 | 351 | C42H62O17 | 22-hydroxyl-glycyrrhizin | Gancao |
| 110 | 28.35 |  |  | 967.4561 |  | C48H72O20 | Rhaoglycyrrhizin | Gancao |
| 111 | 28.73 | 153.1263 | 131 |  |  | C10H16O | Pulegone | Jingjie |
| 112 | 28.88 | 823.4102 | 471,453 | 821.3965 | 351 | C42H62O16 | Glycyrrhizic acid | Gancao |
| 113 | 29.92 | 455.2061 | 303,179 | 453.1917 | 275,177,149,139 | C26H30O7 | Kushenol I/N | Kushen |
| 114 | 30.13 | 823.4102 | 471,453 | 821.3967 | 351 | C42H62O16 | Licorice saponin H2 | Gancao |
| 115 | 30.50 |  |  | 679.4092 | 471 | C36H58O9 | Ziyuglycoside III | Diyu |
| 116 | 30.56 | 823.4102 | 471,453 | 821.3965 | 351 | C42H62O16 | Licorice saponin K2 | Gancao |
| 117 | 30.79 | 825.4292 | 455,437 | 823.4092 | 351 | C42H64O16 | licorice saponin J2 | Gancao |
| 118 | 30.95 | 439.2124 | 303,179 | 437.1966 | 275,261,161 | C26H30O6 | Kuraridine | Kushen |
| 119 | 31.01 | 355.1185 | 299,287 | 353.1038 | 297,284 | C20H18O6 | Licoflavonol | Gancao |
| 120 | 31.41 | 355.1185 | 299 | 353.1038 | 284,125 | C20H18O6 | isoLicoflavonol | Gancao |
| 121 | 31.88 |  |  | 807.4174 | 351,187 | C42H64O15 | Licorice saponin B2 | Gancao |
| 122 | 31.88 |  |  | 807.4174 | 351,187 | C42H64O15 | 22-dehydroxyl-uralsaponin C | Gancao |
| 123 | 32.64 | 439.1739 | 315 | 437.1624 | 313,287,137 | C25H26O7 | Kushenol C | Kushen |
| 124 | 32.66 | 439.1739 | 315 | 437.1624 | 313,287,261,241,176 | C26H30O6 | Isokurarinone | Kushen |
| 125 | 32.83 | 415.2124 | 119 |  |  | C29H50O | β-Sitosterol | Jingjie |
| 126 | 32.83 |  |  | 603.3903 |  | C35H56O8 | Ziyu glycoside II | Diyu |
| 127 | 33.19 | 425.1975 | 289,283,179,165 | 423.1835 | 261,161,137,124 | C25H28O6 | Sophoraflavanone G | Kushen |
| 128 | 33.77 | 353.1028 | 311,299,153 | 351.0881 | 283,199 | C21H20O5 | Gancaonin M | Gancao |
| 129 | 34.77 | 337.1056 | 270,186 | 335.0951 | 319,305 | C20H16O5 | Glabrone | Gancao |
| 130 | 36.02 | 439.2124 | 165 | 437.1966 | 313,287,243,201,175 | C26H30O6 | Kurarinone | Kushen |
| 131 | 36.06 | 371.1860 | 167,123 | 369.1738 | 135 | C22H26O5 | kanozol R | Gancao |
| 132 | 36.71 | 425.1975 | 313,175,139 | 423.1835 | 229,193 | C26H32O5 | kanozol H | Gancao |
| 133 | 37.75 | 425.1933 | 365,191,135 | 423.1835 | 229,193 | C26H32O5 | Licoricidin | Gancao |
| 134 | 38.05 | 423.1809 | 367,311,299 | 421.1655 | 366,309 | C26H30O5 | kanozol J | Gancao |
| 135 | 38.61 | 301.1404 | 149 |  |  | C16H12O6 | Diosmetin | Jingjie |
| 136 | 42.31 |  |  | 455.3504 | 377,277 | C30H48O3 | Ursolic acid | Jingjie |


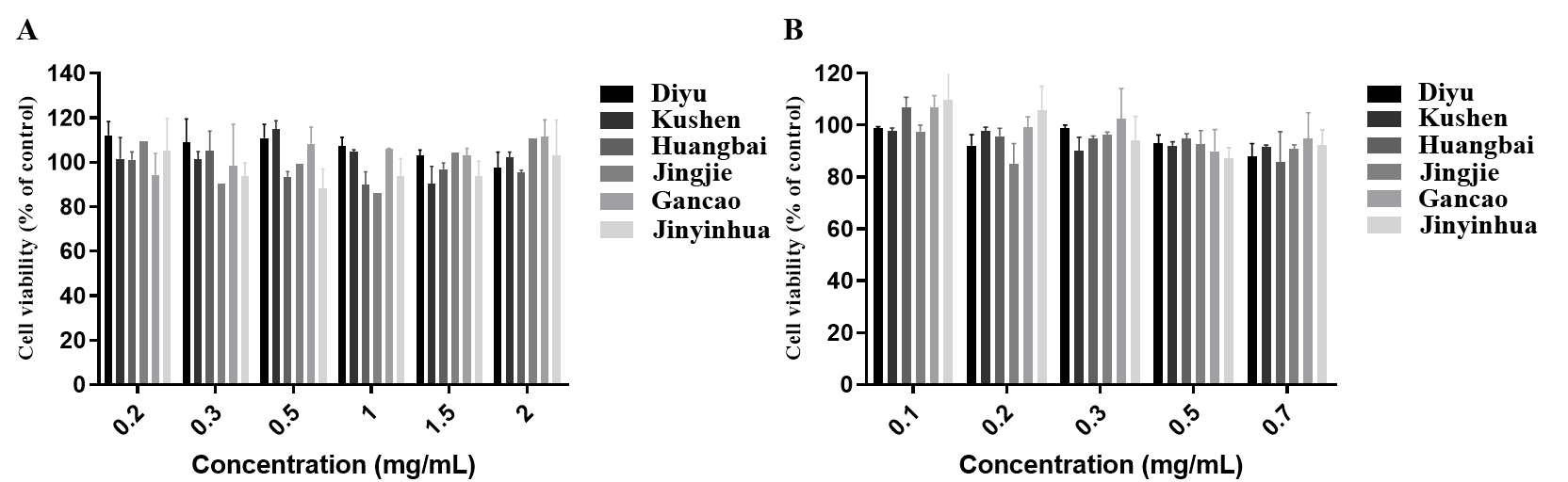


Figure S1. Cytotoxic effects of six single medicinal materials from ‘Eczema mixture’ formula on RBL-2H3 (A) , RAW264.7 (B). The data were analyzed using two-way analysis of variance followed by Turkey’s test.


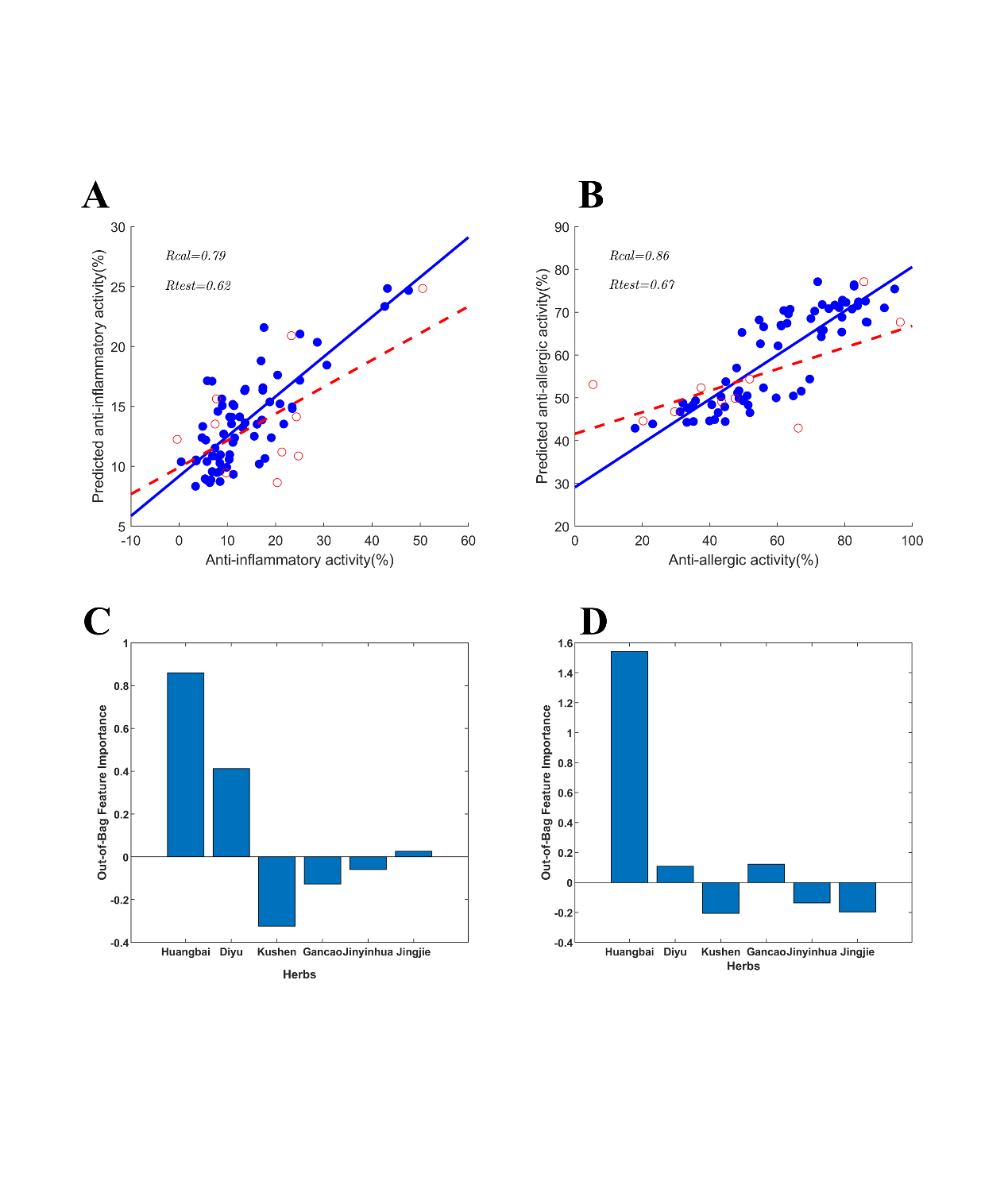


Figure S2. The scatter plot of RF model predicted vs observed values of anti-inflammatory activity (A); the anti-allergic activity (B); the relative importance of each input in determining the anti-inflammatory activity for the six herbs (C) and the anti-allergic activity for the six herbs (D).


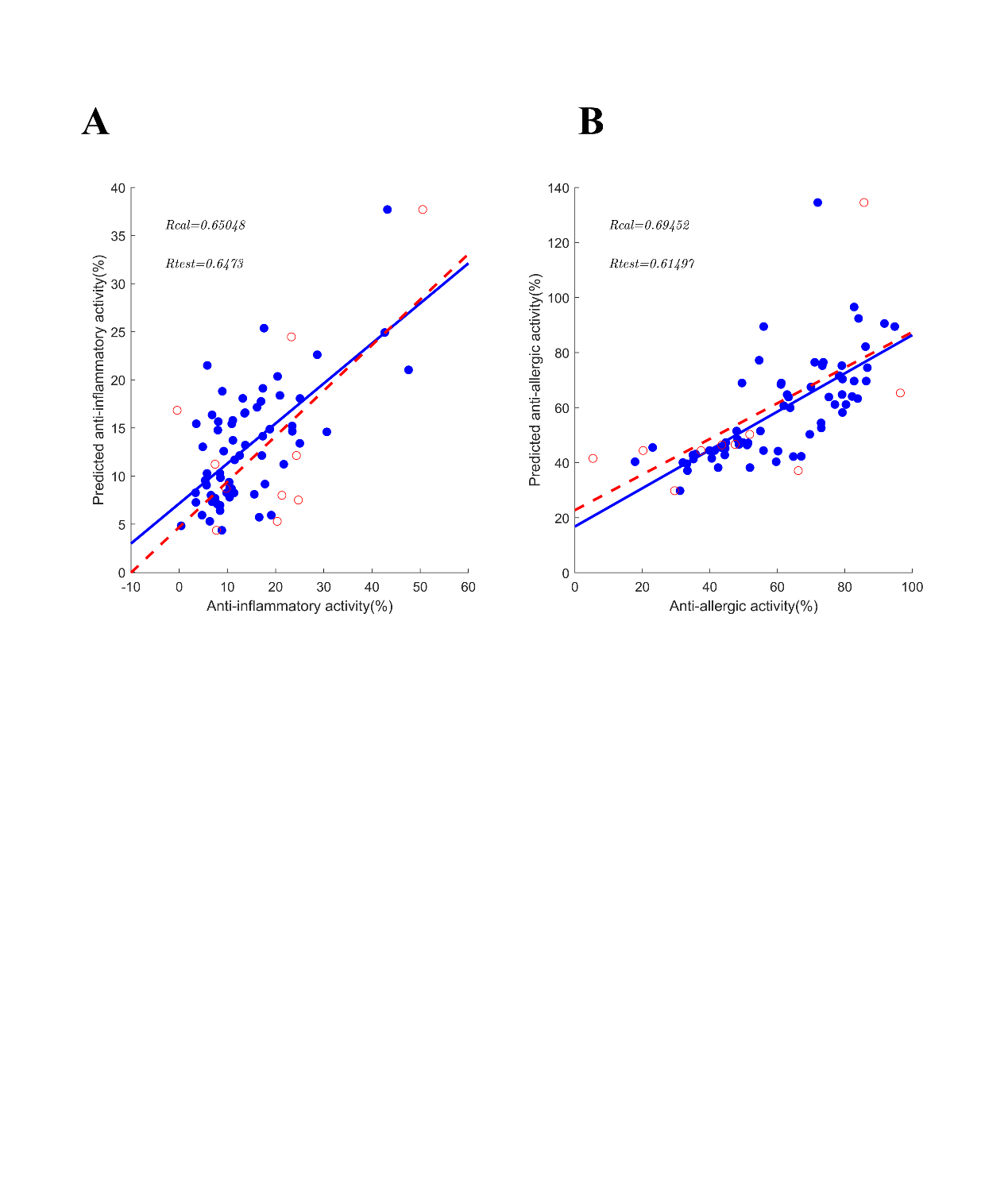


Figure S3. The scatter plot of SVR model predicted vs observed values of anti-inflammatory activity (A); the anti-allergic activity (B).
